# Hip Fracture Treatment and Outcomes Among Community-Dwelling People Living With Dementia

**DOI:** 10.1001/jamanetworkopen.2024.13878

**Published:** 2024-05-30

**Authors:** Rachel R. Adler, Lingwei Xiang, Samir K. Shah, Clancy J. Clark, Zara Cooper, Susan L. Mitchell, Dae Hyun Kim, John Hsu, Karen Sepucha, Richard E. Chunga, Stuart R. Lipsitz, Joel S. Weissman, Andrew J. Schoenfeld

**Affiliations:** 1 Center for Surgery and Public Health, Brigham and Women’s Hospital, Boston, Massachusetts; 2 Division of Vascular Surgery, University of Florida, Gainesville; 3 Division of Surgical Oncology, Wake Forest School of Medicine, Winston-Salem, North Carolina; 4 Hebrew SeniorLife, Marcus Institute for Aging Research, Boston, Massachusetts; 5 Mongan Institute Health Policy Center, Mass General Research Institute, Boston, Massachusetts; 6 Department of Health Care Policy, Harvard Medical School, Boston, Massachusetts; 7 Health Decision Sciences Center, Massachusetts General Hospital, Harvard Medical School, Boston; 8 Department of Orthopedic Surgery, Brigham and Women’s Hospital, Harvard Medical School, Boston, Massachusetts

## Abstract

**Importance:**

The decision for surgical vs nonsurgical treatment for hip fracture can be complicated among community-dwelling people living with dementia.

**Objective:**

To compare outcomes of community-dwelling people living with dementia treated surgically and nonsurgically for hip fracture.

**Design, Setting, and Participants:**

This retrospective cross-sectional study undertook a population-based analysis of national Medicare fee-for-service data. Participants included community-dwelling Medicare beneficiaries with dementia and an inpatient claim for hip fracture from January 1, 2017, to June 30, 2018. Analyses were conducted from November 10, 2022, to October 17, 2023.

**Exposure:**

Surgical vs nonsurgical treatment for hip fracture.

**Main Outcomes and Measures:**

The primary outcome was mortality within 30, 90, and 180 days. Secondary outcomes consisted of selected post–acute care services.

**Results:**

Of 56 209 patients identified with hip fracture (73.0% women; mean [SD] age, 86.4 [7.0] years), 33 142 (59.0%) were treated surgically and 23 067 (41.0%) were treated nonsurgically. Among patients treated surgically, 73.3% had a fracture of the femoral head and neck and 40.2% had moderate to severe dementia (MSD). Among patients with MSD and femoral head and neck fracture, 180-day mortality was 31.8% (surgical treatment) vs 45.7% (nonsurgical treatment). For patients with MSD treated surgically vs nonsurgically, the unadjusted odds ratio (OR) of 180-day mortality was 0.56 (95% CI, 0.49-0.62; *P* < .001) and the adjusted OR was 0.59 (95% CI, 0.53-0.66; *P* < .001). Among patients with mild dementia and femoral head and neck fracture, 180-day mortality was 26.5% (surgical treatment) vs 34.9% (nonsurgical treatment). For patients with mild dementia who were treated surgically vs nonsurgically for femoral head and neck fracture, the unadjusted OR of 180-day mortality was 0.67 (95% CI, 0.60-0.76; *P* < .001) and the adjusted OR was 0.71 (95% CI, 0.63-0.79; *P* < .001). For patients with femoral head and neck fracture, there was no difference in admission to a nursing home within 180 days when treated surgically vs nonsurgically.

**Conclusions and Relevance:**

In this cohort study of community-dwelling patients with dementia and fracture of the femoral head and neck, patients with MSD and mild dementia treated surgically experienced lower odds of death compared with patients treated nonsurgically. Although avoiding nursing home admission is important to persons living with dementia, being treated surgically for hip fracture did not necessarily confer a benefit in that regard. These data can help inform discussions around values and goals with patients and caregivers when determining the optimal treatment approach.

## Key Points

**Question:**

What are the outcomes among community-dwelling people with dementia who are treated surgically compared with nonsurgically for hip fracture?

**Findings:**

In this cross-sectional study of 56 209 community-dwelling patients living with dementia, those treated surgically for fracture of the femoral neck and head experienced reduced mortality; no significant differences were found for other hip fracture locations.

**Meaning:**

These data can help inform discussions around values and goals with patients and caregivers when determining the optimal treatment approach in this population.

## Teaser

This cross-sectional study examines outcomes of surgical compared with nonsurgical treatment of hip fracture by fracture location and dementia severity among community-dwelling patients with dementia.

## Introduction

Hip fracture among older adults often results in decreased independence and quality of life.^1,2^ Surgery is a potential treatment option, yet people living with dementia undergoing this operation experience higher mortality,^2,3,4^ delirium,^2,5^ and postoperative complications,^2,6^ with a consequent greater loss of mobility compared with persons without cognitive difficulties.^2,7^ Much less is known about what happens to patients with hip fractures who do not have surgery. Among patients with advanced dementia residing in nursing homes, one study found that patients who are treated surgically live longer than those who are treated nonsurgically.^8^ However, the majority of people with dementia reside in the community.^9^ Therefore, this study examined the outcomes of surgical treatment compared with nonsurgical treatment of hip fracture among community-dwelling people living with dementia. In line with prior research, we hypothesized that community-dwelling people living with dementia who received surgical intervention for hip fracture would experience superior survival and patient-centered outcomes compared with those managed nonsurgically. To provide further insight into potential impacts on clinical care, we examined outcomes by fracture location and dementia severity. Results from this study may inform treatment decisions around fracture care for people living with dementia, which must balance cognition, mobility, and survival while accounting for baseline cognitive and physical functioning.^10^

## Methods

### Study Design

In this retrospective cross-sectional study of Medicare claims data, we identified community-dwelling people living with dementia who had new hip fracture diagnoses between January 1, 2017, and June 30, 2018, with a look-back period of 1 year. Data were accrued from the following Medicare data files covering claims from January 1, 2016, to December 31, 2018: Master Beneficiary Summary File, Carrier, Inpatient, Outpatient, Skilled Nursing Facility (SNF), Home Health, Outcome and Assessment Information Set, Minimum Data Set (MDS; which tracks patients in SNFs and nursing homes), Durable Medical Equipment, Hospice, and Medicare Data on Physician Practice and Specialty. The Institutional Review Board of Mass General Brigham, Boston, Massachusetts, approved this protocol and granted a waiver of consent for the use of claims data under a data use agreement. We followed the Strengthening the Reporting of Observational Studies in Epidemiology (STROBE) reporting guideline.

### Participants

We used the Inpatient, Outpatient, Carrier, Hospice, Home Health, and SNF files to determine dementia diagnosis, and the Inpatient files to identify hip fracture diagnoses. To identify people living with dementia, we used a validated list of *International Statistical Classification of Diseases, Tenth Revision* (*ICD-10*), diagnosis codes.^11^ To identify hip fractures, we used an established list of *International Classification of Diseases, Ninth Revision* (*ICD-9*), diagnosis codes^8,12^ that were converted to *ICD-10* codes and reviewed by one of the authors (A.J.S.), an orthopedic surgeon. Our algorithm for hip fractures followed best practices to optimize identification of fractures that indicate surgery.^13^ Because surgical and rehabilitation strategies may vary by fracture location, we categorized hip fractures into 4 location types: femoral neck and head, pertrochanteric, subtrochanteric, and multiple (>1). We identified patients treated surgically using an established list of *ICD-10* procedure codes that were converted from *ICD-9* codes^12^ and reviewed by the orthopedic surgeon (A.J.S.). For each patient, the earliest admission with hip fracture diagnosis during the study period was defined as index admission. We considered patients with no MDS assessment in the 180 days before the hip fracture diagnosis to be community-dwelling. The MDS assessment is conducted on admission, at discharge, and at a minimum quarterly during an SNF or a nursing home stay,^14^ and lack of MDS assessment has been used to identify community-dwelling patients.^15^ We included beneficiaries 66 years or older at the index admission and with 6 months of continuous fee-for-service postfracture coverage and 1 year of continuous fee-for-service prefracture coverage, allowing for a 1-month gap in the year prior. We excluded beneficiaries with invalid or missing dates, invalid admission type, or missing urbanicity information from the analysis. A flowchart of cohort selection is found in eFigure 1 in Supplement 1.

### Outcomes

We used the Master Beneficiary Summary File and Inpatient file to identify mortality. In-hospital delirium was identified in Inpatient files using established *ICD-10* diagnosis codes.^16,17^ We defined hospice referral as a new hospice claim in the 180 days after discharge^8^ among patients discharged alive, and home health services as a new home health claim within 10 days after discharge^18^ among patients still alive at 10 days post discharge. Intensive interventions included new dialysis, intubation, resuscitation, mechanical ventilation, or feeding tube insertion^19^ in any Inpatient, SNF, or Outpatient claims any time after hip fracture diagnosis until death or end of the follow-up period. We identified persons admitted to SNF and nursing homes, respectively, using Medicare Parts A and B data to differentiate between SNF stays with rehabilitation services and plans to return to the community compared with nursing home admissions in which persons reside in a nursing home without plans for rehabilitation and return to living in the community.^20^

### Covariates

To provide comparability between the surgical and nonsurgical treatment groups, inverse propensity for treatment weighting (IPTW) for patients were calculated in 8 study subgroups. The subgroups were determined by hip fracture location type and dementia severity^21^ (mild or moderate to severe). The Claims-Based Frailty Index^22^ has been validated to categorize dementia severity^21^ based on Functional Assessment Staging Test stages. In the logistic models to estimate IPTW, we controlled for patient characteristics included in Medicare database files that may have influenced the decision to operate, including admission source, age, dual eligibility status, Elixhauser Comorbidity Index for mortality,^23^ geographic location (metropolitan or nonmetropolitan), race and ethnicity, and sex. Race and ethnicity were included as covariates because they have been linked to risk for surgery. The range for the Elixhauser Comorbidity Index for mortality is −59 to 171, where a higher score indicates more comorbidities present on admission during the index hospitalization.

### Statistical Analysis

Analyses were conducted between November 10, 2022, and October 17, 2023. Continuous data were described using means and SEs, while categorical data were described using frequencies and percentages. Standardized mean differences were used to assess the differences in variables between surgical and nonsurgical treatment groups. An absolute standardized mean difference of 0.10 or less was considered acceptable balance. Unadjusted and adjusted analyses were conducted to examine the associations between treatment groups and outcomes, stratified by hip fracture location and dementia severity. Love plots were generated to compare the balance of covariates between treatment groups before and after IPTW. For ease of display, we present results for head and neck fractures in the Figure and results for other fracture locations in eFigures 2 to 4 in Supplement 1.

**Figure.**
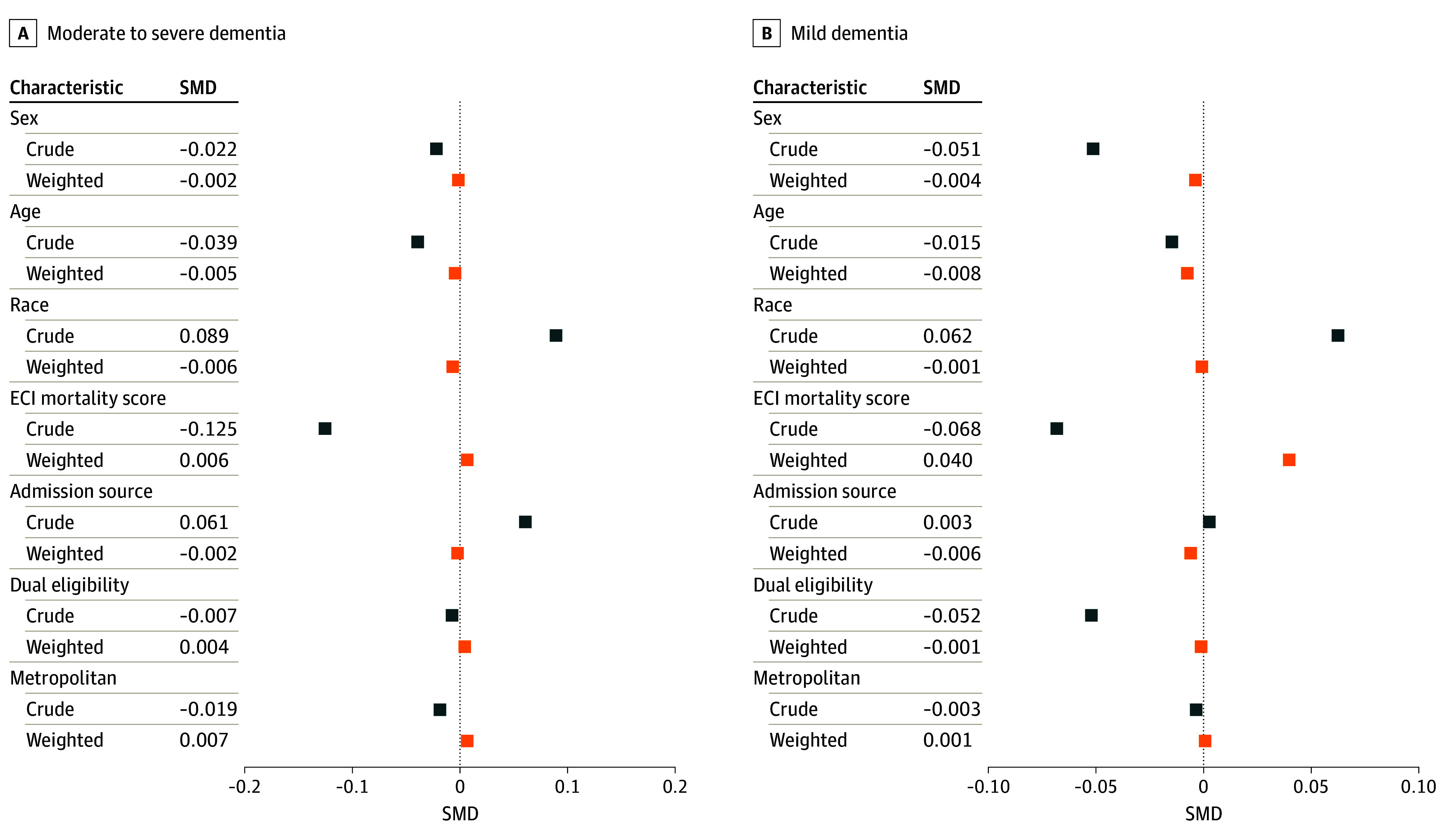
Covariate Balance Before and After Inverse Propensity for Treatment Weighting for Patients With Fracture of the Head and Neck of the Femur The group with moderate to severe dementia included 10 999 community-dwelling people; the group with mild dementia, 16 265 community-dwelling people. SMD indicates standardized mean difference. An absolute SMD of 0.10 or less was considered an acceptable balance of covariates between surgical and nonsurgical treatment groups. ECI indicates Elixhauser Comorbidity Index.

Generalized estimation equations were used to account for clustering within hospitals in examining the unadjusted and adjusted associations between surgical treatment and binary outcomes of mortality, in-hospital delirium, new hospice referral, and new home health services use. Kaplan-Meier curves and IPTW Kaplan-Meier curves,^24^ accounting for death as a competing risk, were calculated to obtain the unadjusted and adjusted proportions of intensive interventions,^19^ SNF admission,^20^ and nursing home admission^20^ at 3 points (30, 90, and 180 days) after discharge from the index admission (maximum follow-up time, 180 days). To provide a more interpretable outcome, we conducted survival analysis first, and then instead of hazard ratios, we reported the probabilities (cumulative incidence function estimates) of outcomes within 30, 90, and 180 days by extracting numbers from a cumulative incidence table derived from survival analysis using the BASELINE statement in SAS. Because our analyses were based on preplanned hypotheses, we did not adjust the *P* values^25^ and therefore considered a 2-sided *P* < .05 to be statistically significant. All data and Love plots were analyzed using SAS, version 9.4 (SAS Institute Inc).

## Results

We identified 56 209 community-dwelling individuals with dementia who had a hip fracture diagnosis in Inpatient claims during the study period. The mean (SD) age of the cohort was 86.4 (7.0) years; 73.0% were women, and 27.0% were men. In terms of race and ethnicity, 2.2% of the individuals were Alaska Native, American Indian, or Asian; 4.2% were Hispanic; 4.0% were non-Hispanic Black; 89.0% were non-Hispanic White; and 0.6% were of other (ie, not further identified) or unknown race or ethnicity. Among the study population, 33 142 (59.0%) were treated surgically, and 23 067 (41.0%) received nonsurgical treatment. Among patients treated surgically, most had a femoral head and neck fracture (73.3%), while most patients treated nonsurgically had a pertrochanteric fracture (78.5%). Among patients who were treated surgically, 40.2% had MSD, whereas 42.8% who were treated nonsurgically had MSD. The Elixhauser Comorbidity Index mortality score was similar in each group, with a mean (SD) of 12.4 (15.8) among patients who were treated nonsurgically and 11.6 (15.1) among patients who were treated surgically (Table 1). The Figure shows the covariate balance before and after IPTW for patients with femoral head and neck fracture; eFigures 2 to 4 in Supplement 1 show the covariate balance before and after IPTW for patients with pertrochanteric, subtrochanteric, and multiple fracture locations. eTable 1 in Supplement 1 reports the crude and weighted standardized mean difference for each fracture location.

**Table 1.** Characteristics of Community-Dwelling People Living With Dementia Who Had Hip Fracture Treated Surgically vs Nonsurgically

Characteristic	Treatment group^a^			SMD
	All (N = 56 209)	Nonsurgical (n = 23 067)	Surgical (n = 33 142)	
Age, mean (SD), y	86.4 (7.0)	86.8 (7.0)	86.1 (7.0)	−0.10
Sex				
Women	41 028 (73.0)	17 066 (74.0)	23 962 (72.3)	0.04
Men	15 181 (27.0)	6001 (26.0)	9180 (27.7)	
Race, No. (%)				
Alaska Native, American Indian, or Asian	1218 (2.2)	520 (2.3)	698 (2.1)	0.15
Hispanic	2369 (4.2)	1111 (4.8)	1258 (3.8)	
Non-Hispanic Black	2268 (4.0)	854 (3.7)	1414 (4.3)	
Non-Hispanic White	50 023 (89.0)	20 454 (88.7)	29 569 (89.2)	
Other^b^	242 (0.4)	88 (0.4)	154 (0.5)	
Unknown	89 (0.2)	40 (0.2)	49 (0.1)	
ECI for in-hospital mortality, mean (SD)^c^	11.9 (15.4)	12.4 (15.8)	11.6 (15.1)	−0.04
Comorbidities				
Cerebrovascular disease	3858 (6.9)	1620 (7.0)	2238 (6.8)	−0.01
Congestive heart failure	11 055 (19.7)	4739 (20.5)	6316 (19.1)	−0.04
Diabetes with chronic complications	9293 (16.5)	3990 (17.3)	5303 (16.0)	−0.03
Diabetes without chronic complications	12 403 (22.1)	5247 (22.7)	7156 (21.6)	−0.03
Hypertension, complicated	21 084 (37.5)	8895 (38.6)	12 189 (36.8)	−0.04
Hypertension, uncomplicated	44 250 (78.7)	18 316 (79.4)	25 934 (78.3)	−0.03
Chronic pulmonary disease	14 139 (25.2)	5944 (25.8)	8195 (24.7)	−0.02
Neurological disorders affecting movement	4359 (7.8)	1583 (6.9)	2776 (8.4)	0.06
Other neurological disorders	6711 (11.9)	2823 (12.2)	3888 (11.7)	−0.02
Peripheral vascular disease	13 246 (23.6)	5703 (24.7)	7543 (22.8)	−0.05
Kidney failure, moderate	10 692 (19.0)	4375 (19.0)	6317 (19.1)	0.00
Kidney failure, severe	2411 (4.3)	1027 (4.5)	1384 (4.2)	−0.01
Weight loss	7278 (12.9)	3210 (13.9)	4068 (12.3)	−0.05
Fracture location^d^				
Fracture of head and neck of femur (S72.0)	27 253 (48.5)	2973 (12.9)	24 280 (73.3)	1.53
Pertrochanteric fracture (S72.1)	25 824 (45.9)	18 103 (78.5)	7721 (23.3)	
Subtrochanteric fracture (S72.2)	1793 (3.2)	1232 (5.3)	561 (1.7)	
Multiple locations	1339 (2.4)	759 (3.3)	580 (1.8)	
Treatment type				
Internal fixation only	13 456 (23.9)	0	13 456 (40.6)	9.90
Internal fixation and hemiarthroplasty	386 (0.7)	0	386 (1.2)	
Internal fixation and total hip arthroplasty	27 (0.0)	0	27 (0.1)	
Hemiarthroplasty only	18 605 (33.1)	0	18 605 (56.1)	
Hemiarthroplasty and total hip arthroplasty	NA^e^	0	NA^e^	
Total hip arthroplasty only	661 (1.2)	0	661 (2.0)	
All procedures	NA^e^	0	NA^e^	
Nonoperative	23 067 (41.0)	23 067 (100)	0	
Index hip fracture admitting source				
Home	49 132 (87.4)	20 174 (87.5)	28 958 (87.4)	<0.001
Facility	3685 (6.6)	1521 (6.6)	2164 (6.5)	
Hospital transfers	3241 (5.8)	1311 (5.7)	1930 (5.8)	
Hospice	11 (0.0)	NA^e^	NA^e^	
Unknown	140 (0.2)	NA^e^	NA^e^	
Index hip fracture discharge status				
Home self-care	1248 (2.2)	561 (2.4)	687 (2.1)	0.19
Home with home health agency care	2393 (4.3)	1012 (4.4)	1381 (4.2)	
SNF	40 187 (71.5)	15 976 (69.3)	24 211 (73.1)	
Nursing home	88 (0.2)	41 (0.2)	47 (0.1)	
Inpatient rehabilitation	6629 (11.8)	2627 (11.4)	4002 (12.1)	
Other hospital	1316 (2.3)	562 (2.4)	754 (2.3)	
Hospice	2306 (4.1)	1268 (5.5)	1038 (3.1)	
Death	1428 (2.5)	760 (3.3)	668 (2.0)	
Other	614 (1.1)	260 (1.1)	354 (1.1)	
Claims-Based Frailty Index^f^				
≥0.28	23 185 (41.2)	9865 (42.8)	13 320 (40.2)	−0.05
<0.28	33 024 (58.8)	13 202 (57.2)	19 822 (59.8)	
Urbanicity				
Metropolitan	43 680 (77.7)	18 170 (78.8)	25 510 (77.0)	−0.04
Nonmetropolitan	12 529 (22.3)	4897 (21.2)	7632 (23.0)	

Abbreviations: ECI, Elixhauser Comorbidity Index; NA, not available; SMD, standardized mean difference; SNF, skilled nursing facility.

^a^ Unless otherwise indicated, data are expressed as No. (%). Percentages have been rounded and may not total 100.

^b^ Unknown because the Research Data Assistance Center does not provide that information in their data dictionaries.

^c^ Scores range from −59 to 171, with a higher score indicating more comorbidities present on admission during the index hospitalization.

^d^ Data in parentheses indicate codes from the *International Statistical Classification of Diseases, Tenth Revision.*

^e^ Data suppressed according to Medicare data policies.

^f^ Scores of 0.28 or greater indicate moderate to severe dementia; less than 0.28, mild dementia.

### Mortality

Among people living with dementia who had femoral head and neck fracture, surgery was associated with lower unadjusted odds of death compared with no surgery for patients with both MSD and mild dementia at 30, 90, and 180 days after hip fracture diagnosis (Table 2), with 180-day mortality of 31.8% of those receiving surgical treatment compared with 45.7% for those receiving nonsurgical treatment (unadjusted odds ratio [OR], 0.56 [95% CI, 0.49-0.62]; *P* < .001). Among patients with mild dementia and femoral head and neck fracture, 180-day mortality was 26.5% for those receiving surgical treatment vs 34.9% for those receiving nonsurgical treatment (unadjusted OR, 0.67 [95% CI, 0.60-0.76]; *P* < .001). After applying the IPTW model for adjustment, relative odds of death for people living with dementia treated surgically vs nonsurgically remained lower for both groups at 30 days (adjusted OR [AOR] for MSD, 0.33 [95% CI, 0.29-0.38; *P* < .001]; AOR for mild dementia, 0.39 [95% CI, 0.34-0.45; *P* < .001]), 90 days (AOR for MSD, 0.50 [95% CI, 0.44-0.56; *P* < .001]; AOR for mild dementia, 0.60 [95% CI, 0.53-0.67; *P* < .001]), and 180 days (AOR for MSD, 0.59 [95% CI, 0.53-0.66; *P* < .001]; AOR for mild dementia, 0.71 [95% CI, 0.63-0.79; *P* < .001]) (Table 2). There were few differences in mortality for patients treated surgically vs nonsurgically for pertrochanteric, subtrochanteric, or multiple fracture locations (eTables 2 and 3 in Supplement 1).

**Table 2.** Likelihood of Outcomes of Community-Dwelling People With Dementia Treated Surgically vs Nonsurgically for Fracture of the Head and Neck of Femur by Dementia Severity

Outcome by dementia severity^a^	No. of patients	Unadjusted likelihood					Adjusted likelihood^c^			
		Treatment group, No. (%)^b^		OR (95% CI)	*P* value	Treatment group, No. (%)			OR (95% CI)	*P* value
		Nonsurgical	Surgical			Nonsurgical		Surgical		
**Death**										
Within 30 d										
MSD	10 988	380 (28.0)	1043 (10.8)	0.31 (0.27-0.36)	<.001	369 (27.1)		1048 (10.9)	0.33 (0.29-0.38)	<.001
Mild	16 265	319 (19.8)	1216 (8.3)	0.37 (0.32-0.42)	<.001	306 (19.0)		1221 (8.3)	0.39 (0.34-0.45)	<.001
Within 90 d										
MSD	10 988	513 (37.8)	2148 (22.3)	0.47 (0.42-0.53)	<.001	499 (36.7)		2158 (22.4)	0.50 (0.44-0.56)	<.001
Mild	16 265	459 (28.4)	2685 (18.3)	0.57 (0.50-0.64)	<.001	443 (27.4)		2692 (18.4)	0.60 (0.53-0.67)	<.001
Within 180 d										
MSD	10 988	620 (45.7)	3063 (31.8)	0.56 (0.49-0.62)	<.001	602 (44.3)		3075 (31.9)	0.59 (0.53-0.66)	<.001
Mild	16 265	563 (34.9)	3879 (26.5)	0.67 (0.60-0.76)	<.001	547 (33.9)		3888 (26.5)	0.71 (0.63-0.79)	<.001
**In-hospital delirium**										
MSD	10 988	227 (16.7)	1787 (18.6)	1.14 (0.97-1.33)	.11	214 (15.7)		1800 (18.7)	1.23 (1.06-1.44)	.008
Mild	16 265	243 (15.0)	2242 (15.3)	1.02 (0.89-1.17)	.78	225 (13.9)		2257 (15.4)	1.12 (0.97-1.30)	.11
**Hospice referral** ^d^										
MSD	10 652	476 (38.1)	2749 (29.2)	0.67 (0.59-0.76)	<.001	471 (37.4)		2755 (29.3)	0.69 (0.61-0.79)	<.001
Mild	15 961	410 (26.8)	3398 (23.6)	0.84 (0.74-0.95)	.007	405 (26.3)		3400 (23.6)	0.86 (0.77-0.97)	.02
**Home health services** ^e^										
MSD	10 036	70 (6.6)	480 (5.4)	0.80 (0.61-1.04)	.10	71 (6.6)		480 (5.4)	0.80 (0.62-1.04)	.10
Mild	15 333	122 (8.8)	974 (7.0)	0.78 (0.64-0.95)	.01	123 (8.8)		972 (7.0)	0.78 (0.64-0.95)	.01

Abbreviations: MSD, moderate to severe dementia; OR, odds ratio.

^a^ A Claims-Based Frailty Index of less than 0.28 indicated mild dementia; 0.28 or greater, MSD.

^b^ Includes 1358 patients with MSD and 1615 with mild dementia in the nonsurgical group and 9630 with MSD and 14 650 with mild dementia in the surgical group.

^c^ Calculated using inverse propensity-weighted regression. For each hip fracture location and Claims-Based Frailty Index severity group combination, propensity scores for the management groups were calculated by age, sex, race and ethnicity, Elixhauser Comorbidity Index mortality score, admission source, urbanicity, and dual eligibility.

^d^ Restricted to patients who were discharged alive.

^e^ Restricted to patients who were discharged alive and did not die within 10 days after discharge.

### Delirium, Hospice, and Home Health Services

In patients with femoral head and neck fractures, surgery was associated with higher odds of in-hospital delirium (AOR, 1.23 [95% CI, 1.06-1.44]; *P* = .008) compared with no surgery only for those with MSD (Table 2); there were no significant differences for in-hospital delirium among patients with mild dementia (Table 2) or other fracture locations (eTables 2 and 3 in Supplement 1). Patients with both MSD and mild dementia who were treated surgically for femoral head and neck fracture had lower odds of hospice referral compared with those treated nonsurgically (AOR for MSD, 0.69 [95% CI, 0.61-0.79; *P* < .001]; AOR for mild dementia, 0.86 [95% CI, 0.77-0.97; *P* = .02]) (Table 2). Patients with mild dementia who were treated surgically vs nonsurgically for pertrochanteric and subtrochanteric fractures also had lower odds of hospice referral (eTable 3 in Supplement 1). Additionally, only patients with mild dementia who were treated surgically vs nonsurgically for femoral head and neck fracture had lower odds of new home health services use (AOR, 0.78 [95% CI, 0.64-0.95]; *P* = .01). There were no differences in new home health services use for patients with MSD who were treated surgically vs nonsurgically for femoral head and neck fracture (Table 2).

### Intensive Interventions, SNF Admission, and Nursing Home Admission

In models adjusting for competing risk of death, there were no significant differences in intensive interventions for patients with MSD or mild dementia who were treated surgically vs nonsurgically for any fracture location (Table 3 and eTable 4 in Supplement 1). The risk of admission to an SNF for rehabilitation services within 90 days was significantly higher for people living with dementia who were treated surgically vs nonsurgically for femoral head and neck fractures in both the MSD (surgical management, 66% [95% CI, 65%-67%]; nonsurgical management, 51% [95% CI, 50%-53%]; *P* < .001) and mild dementia groups (surgical management, 6% [95% CI, 65%-66%]; nonsurgical management, 58% [95% CI, 56%-60%]; *P* < .001) (Table 3); however, there were no significant differences for admission to a nursing home with no rehabilitation services for people living with dementia who were treated surgically vs nonsurgically by dementia severity or any hip fracture location subgroup (Table 3 and eTable 4 in Supplement 1).

**Table 3.** Likelihood of Outcomes of Community-Dwelling People With Dementia Treated Surgically vs Nonsurgically for Fracture of the Head and Neck of Femur by Dementia Severity, Among Those Discharged Alive

Outcome by dementia severity^a^	No. of patients	Unadjusted likelihood			Adjusted likelihood^b^		
		Treatment group, % (95% CI)		*P* value	Treatment group, % (95% CI)		*P* value
		Nonsurgical (n = 2782)	Surgical (n = 23 831)		Nonsurgical	Surgical	
**Intensive interventions**							
Within 30 d							
MSD	10 652	4 (3-5)	4 (4-5)	.94	4 (3-5)	4 (4-5)	.39
Mild	15 961	3 (2-3)	2 (2-3)	.93	2 (2-3)	2 (2-3)	.49
Within 90 d							
MSD	10 652	6 (5-7)	6 (5-6)	.94	5 (4-7)	6 (5-6)	.39
Mild	15 961	4 (3-5)	3 (3-4)	.93	3 (2-4)	3 (3-4)	.49
Within 180 d							
MSD	10 652	NA^c^	7 (6-7)	.94	NA^c^	7 (6-7)	.39
Mild	15 961	NA^c^	NA^c^	.93	NA^c^	NA^c^	.49
**Admission to SNF**							
Within 30 d							
MSD	10 652	49 (48-51)	65 (64-66)	<.001	50 (48-51)	65 (64-65)	<.001
Mild	15 961	56 (55-58)	64 (63-64)	<.001	56 (55-58)	64 (63-64)	<.001
Within 90 d							
MSD	10 652	51 (49-53)	66 (65-67)	<.001	51 (50-53)	66 (65-67)	<.001
Mild	15 961	58 (56-60)	65 (64-66)	<.001	58 (56-60)	65 (65-66)	<.001
Within 180 d							
MSD	10 652	NA^c^	67 (66-68)	<.001	NA^c^	67 (66-68)	<.001
Mild	15 961	NA^c^	NA^c^	<.001	NA^c^	NA^c^	<.001
**Admission to nursing home**							
Within 30 d							
MSD	10 652	20 (19-22)	22 (21-22)	.17	21 (19-22)	22 (21-22)	.23
Mild	15 961	21 (20-22)	20 (20-20)	.98	21 (20-22)	20 (20-20)	.67
Within 90 d							
MSD	10 652	24 (23-26)	25 (24-26)	.17	24 (23-26)	25 (24-26)	.23
Mild	15 961	23 (22-24)	23 (23-23)	.98	23 (22-24)	23 (23-24)	.67
Within 180 d							
MSD	10 652	NA^c^	27 (27-28)	.17	NA^c^	27 (27-28)	.23
Mild	15 961	NA^c^	25 (25-25)	.98	NA^c^	25 (25-25)	.67
**Readmission to acute care facility**							
Within 30 d							
MSD	10 652	14 (13-16)	17 (16-18)	<.001	14 (12-16)	17 (16-18)	<.001
Mild	15 961	15 (14-17)	13 (13-14)	.40	15 (13-17)	13 (13-14)	.77
Within 90 d							
MSD	10 652	25 (23-27)	31 (30-32)	<.001	25 (23-27)	31 (30-32)	<.001
Mild	15 961	25 (23-27)	24 (23-25)	.40	24 (22-27)	24 (23-25)	.77
Within 180 d							
MSD	10 652	33 (30-35)	40 (39-40)	<.001	33 (30-35)	40 (39-41)	<.001
Mild	15 961	33 (30-35)	32 (31-33)	.40	32 (30-35)	32 (31-33)	.77

Abbreviations: MSD, moderate to severe dementia; NA, not available.

^a^ A Claims-Based Frailty Index of less than 0.28 indicated mild dementia; 0.28 or greater, MSD.

^b^ Calculated using inverse propensity-weighted regression. For each hip fracture location and Claims-Based Frailty Index severity group combination, propensity scores for the management groups were calculated by age, sex, race and ethnicity, Elixhauser Comorbidity Index mortality score, admission source, urbanicity, and dual eligibility.

^c^ No outcome event was observed on or after the last day of the follow-up period.

## Discussion

In this large national cross-sectional study of community-dwelling people living with dementia who had hip fracture, we found that 59.0% of people living with dementia with hip fracture were treated surgically and 41.0% were treated nonsurgically. Surgical treatment and outcomes varied by fracture location and dementia severity. Surgical treatment of femoral head and neck fractures were associated with reduced mortality and fewer hospice referrals for patients, regardless of dementia severity, but was not associated with improved outcomes for hip fracture location.

Previous studies have reported varying proportions of nonsurgical treatment for hip fracture in similar populations, ranging among 8.1% of patients with advanced dementia^26^ to 10.6% of older adults,^27^ 11.8% of nursing home residents,^28^ and 15.2% of nursing home residents with advanced dementia.^8^ Though the present study found a greater proportion of people living with dementia who were receiving nonsurgical management (41.0%) than previous studies, it aligns with prior work highlighting a higher prevalence of dementia among patients treated nonsurgically for hip fracture.^27,29^ In a retrospective study analyzing outcomes of adults older than 70 years, Ishimaru et al^29^ reported that 50% of patients treated nonsurgically for hip fractures (femoral neck and pertrochanteric fractures) had dementia compared with 38.2% of patients treated surgically at a single institution. In a similar study of patients older than 65 years who were treated surgically vs nonsurgically for hip fracture (femoral head and neck and pertrochanteric fractures), Jain et al^27^ used a population database supplemented with hospital record review and found that 35.5% of the group treated nonsurgically had dementia compared with 25.9% of the group treated surgically. Given these previous findings and the fact that the present study is limited to people living with dementia, it is not surprising that a relatively high proportion of our cohort received nonsurgical management.

Our findings align with and extend previous research indicating lower mortality among older adults treated for hip fracture surgically,^28,30^ including among nursing home residents with advanced dementia.^8^ The findings of the present study suggest that surgery was associated with reduced mortality compared with nonsurgical treatment, but only among community-dwelling people living with dementia who had fracture of the femoral head and neck. This was consistent for both patients with MSD and mild dementia. These findings are in line with those of Berry et al,^8^ who reported reduced mortality among nursing home residents with advanced dementia treated surgically for hip fracture compared with patients treated nonsurgically. Interestingly, we found little difference in mortality between patients treated surgically vs nonsurgically for other hip fracture locations. This may stem from differing levels of surgical intensity that may vary depending on the location of hip fracture (eg, femoral head and neck vs pertrochantric) and differences in postoperative rehabilitation strategies, which may contribute to higher risks of complications and other adverse events.^13,31,32^ Femoral head and neck fractures are often treated by arthroplasty, which results in more predictable mobility and thus may have contributed to improved rehabilitation and lower mortality in our cohort.

Delirium is an important outcome because it affects a patient’s quality of life^33^ and ability to engage in rehabilitation to improve function and mobility following hip fracture. We found odds of delirium higher for patients with MSD treated surgically vs nonsurgically for fractures of the femoral head and neck, but no differences in delirium for patients with mild dementia or those treated surgically vs nonsurgically with other fracture locations. Delirium in post–acute care settings is associated with poorer functional status recovery^34^ and may limit a person’s ability to engage in rehabilitation. These are important considerations given that our study found that more patients who were treated surgically vs nonsurgically were sent to a skilled nursing facility for rehabilitation after the index hospitalization.

Our findings suggest that surgery among people living with dementia who had femoral head and neck fractures was associated with reduced mortality, regardless of dementia severity. When treated surgically, these patients experienced fewer hospice referrals through the end of the study period, and patients with mild dementia experienced less new use of home health services within 10 days of discharge. However, we found little to no survival benefit following surgery or changes among other outcomes among people living with dementia who had hip fractures in other locations. Additionally, we found no difference in nursing home admission (compared with SNF admission) among people living with dementia treated surgically vs nonsurgically for hip fractures in any location, regardless of dementia severity. People living with dementia typically want to remain in the community for as long as possible.^35,36^ Although many persons treated surgically for hip fracture must transition to SNFs temporarily for rehabilitation purposes, our findings suggest that being treated surgically for hip fracture does not necessarily confer a benefit in terms of avoiding nursing home admission, where rehabilitation services are not offered with the plan for the resident to transition back to living in the community.

### Strengths and Limitations

Strengths of this study include the large national sample and focus on community-dwelling people living with dementia. Comparing surgical outcomes between people living with dementia and people without dementia is informative but may not provide the data needed for surgical decision-making for people living with dementia, given that clinicians often expect people living with dementia to have poorer outcomes due to the high-risk nature of the patient, and not the procedure. By focusing our analyses solely on outcomes among people living with dementia treated surgically vs nonsurgically for hip fracture and examining differences by fracture location and dementia severity, we provide important information that can be used by people living with dementia, their caregivers, and their clinicians to support shared decision-making regarding optimal care in the period immediately following a hip fracture.

This study has some limitations. First, we relied on administrative data, which has the potential for confounding from unmeasured variables. While data from medical records often contain more clinical context, these data likewise have inherent limitations, including incomplete or missing records, lack of standardized interpretation of structured fields, data stored in unretrievable notes, and lack of national perspective as well as lack of standardized recording practices across health systems.^37^ Second, we were unable to assess individual functional status or mobility in relation to treatment strategy or outcomes, which may have influenced management choices or changed as an outcome of the treatment; however, we attempted to mitigate this limitation by incorporating the Claims-Based Frailty Index, which has been validated against physical performance and activities of daily living dependence.^38,39^ Third, we were unable to examine recovery time, engagement in rehabilitation, or quality of life. Last, we do not have information about care preferences or goals of care, which may have influenced both decisions about surgery and certain outcomes, such as hospice.

## Conclusions

In this cohort study of community-dwelling Medicare beneficiaries living with dementia, surgical treatment and outcomes varied by fracture location, with a clear benefit of surgery for people living with dementia who had femoral head and neck fracture and few differences in outcomes between surgical and nonsurgical treatment among other types of hip fractures. These data can help inform discussions around values and goals with patients and caregivers when determining the optimal treatment approach in this population.

## Notes

Supplement 1. **eFigure 1.** Cohort Selection Flowchart **eFigure 2.** Covariate Balance Before and After IPTW for Patients With Pertrochanteric Fracture **eFigure 3.** Covariate Balance Before and After IPTW for Patients With Subtrochanteric Fracture **eFigure 4.** Covariate Balance Before and After IPTW for Patients With Fractures in Multiple Locations **eTable 1.** Standardized Mean Difference for Crude and IPTW Data by Fracture Location **eTable 2.** Unadjusted and Adjusted Likelihood of Outcomes of Community-Dwelling People With Moderate to Severe Dementia Treated Surgically vs Nonsurgically, by Hip Fracture Location **eTable 3.** Unadjusted and Adjusted Likelihood of Outcomes of Community-Dwelling People Mild With Dementia Treated Surgically vs Nonsurgically, by Hip Fracture Location **eTable 4.** Unadjusted and Adjusted Likelihood of Outcomes of Community-Dwelling People With Dementia Treated Surgically vs Nonsurgically, by Hip Fracture Location and Dementia Severity
eReference

Supplement 2.
Data Sharing Statement
