# Supplementary material for: Hip Fracture Treatment and Outcomes Among Community-Dwelling People Living With Dementia
Source: JAMA Netw Open. 2024 May 30;7(5):e2413878. doi: 10.1001/jamanetworkopen.2024.13878 (PMC11140536; doi:10.1001/jamanetworkopen.2024.13878)
Supplement: Supplement 1. — eFigure 1. Cohort Selection Flowchart eFigure 2. Covariate Balance Before and After IPTW for Patients With Pertrochanteric Fracture eFigure 3. Covariate Balance Before and After IPTW for Patients With Subtrochanteric Fracture eFigure 4. Covariate Balance Before and After IPTW for Patients With Fractures in Multiple Locations eTable 1. Standardized Mean Difference for Crude and IPTW Data by Fracture Location eTable 2. Unadjusted and Adjusted Likelihood of Outcomes of Community-Dwelling People With Moderate to Severe Dementia Treated Surgically vs Nonsurgically, by Hip Fracture Location eTable 3. Unadjusted and Adjusted Likelihood of Outcomes of Community-Dwelling People Mild With Dementia Treated Surgically vs Nonsurgically, by Hip Fracture Location eTable 4. Unadjusted and Adjusted Likelihood of Outcomes of Community-Dwelling People With Dementia Treated Surgically vs Nonsurgically, by Hip Fracture Location and Dementia Severity eReference [file jamanetwopen-e2413878-s001.pdf]

Supplementary Online Content

Adler RR, Xiang L, Shah SK, et al. Hip Fracture treatment and outcomes among community-dwelling people living with dementia. *JAMA Netw Open*. 2024;7(5):e2413878. doi:10.1001/jamanetworkopen.2024.13878

eFigure 1. Cohort Selection Flowchart

eFigure 2. Covariate Balance Before and After IPTW for Patients With Pertrochanteric Fracture

eFigure 3. Covariate Balance Before and After IPTW for Patients With Subtrochanteric Fracture

eFigure 4. Covariate Balance Before and After IPTW for Patients With Fractures in Multiple Locations

eTable 1. Standardized Mean Difference for Crude and IPTW Data by Fracture Location

eTable 2. Unadjusted and Adjusted Likelihood of Outcomes of Community-Dwelling People With Moderate to Severe Dementia Treated Surgically vs Nonsurgically, by Hip Fracture Location

eTable 3. Unadjusted and Adjusted Likelihood of Outcomes of Community-Dwelling People Mild With Dementia Treated Surgically vs Nonsurgically, by Hip Fracture Location

eTable 4. Unadjusted and Adjusted Likelihood of Outcomes of Community-Dwelling People with Dementia Treated Surgically vs Nonsurgically, by Hip Fracture Location and Dementia Severity

eReference

This supplementary material has been provided by the authors to give readers additional information about their work.

© 2024 Adler RR et al. *JAMA Network Open*.

eFigure 1. Cohort Selection Flowchart

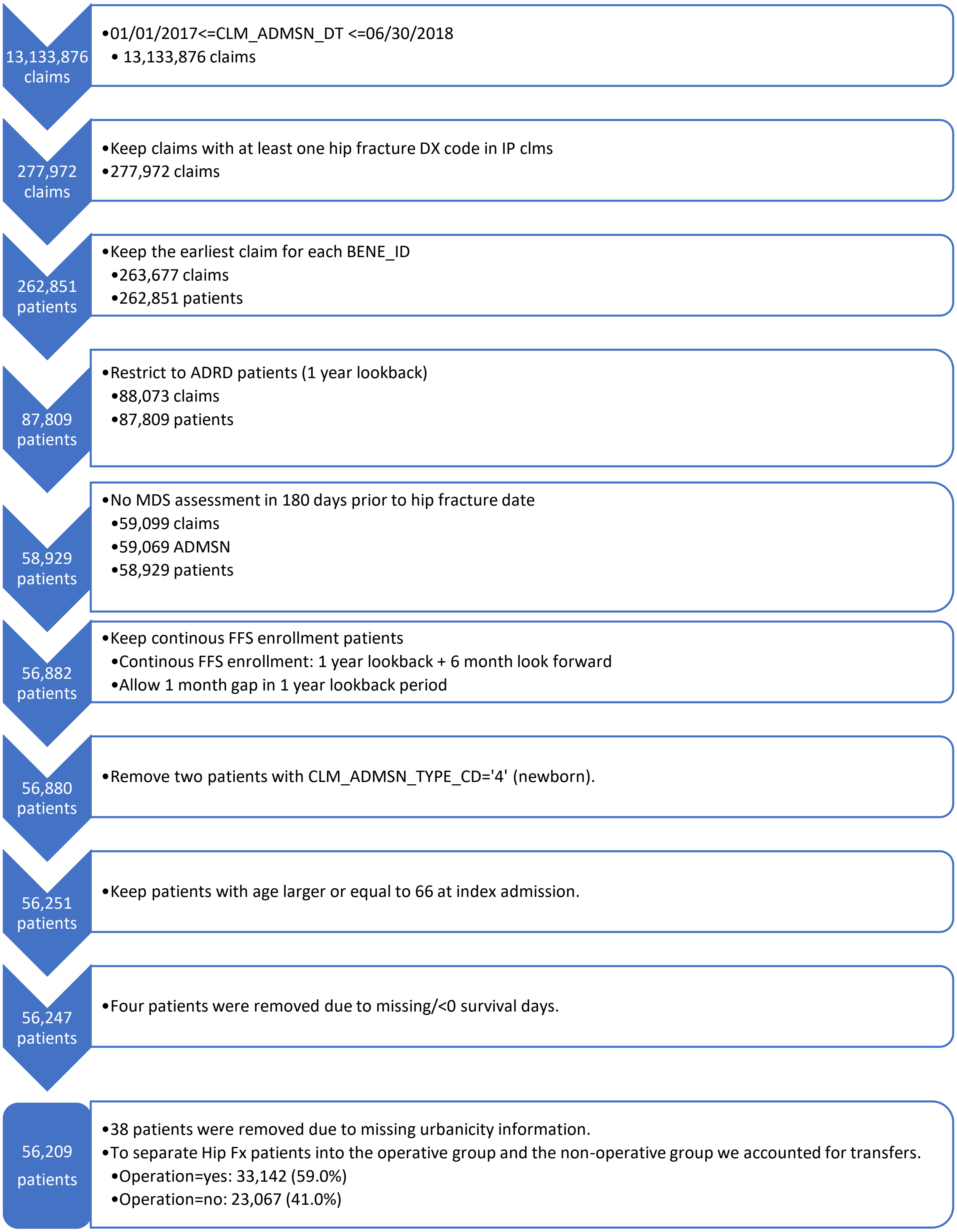

© 2024 Adler RR et al. *JAMA Network Open*.

eFigure 2. Covariate Balance Before and After IPTW for Patients With Pertrochanteric Fracture

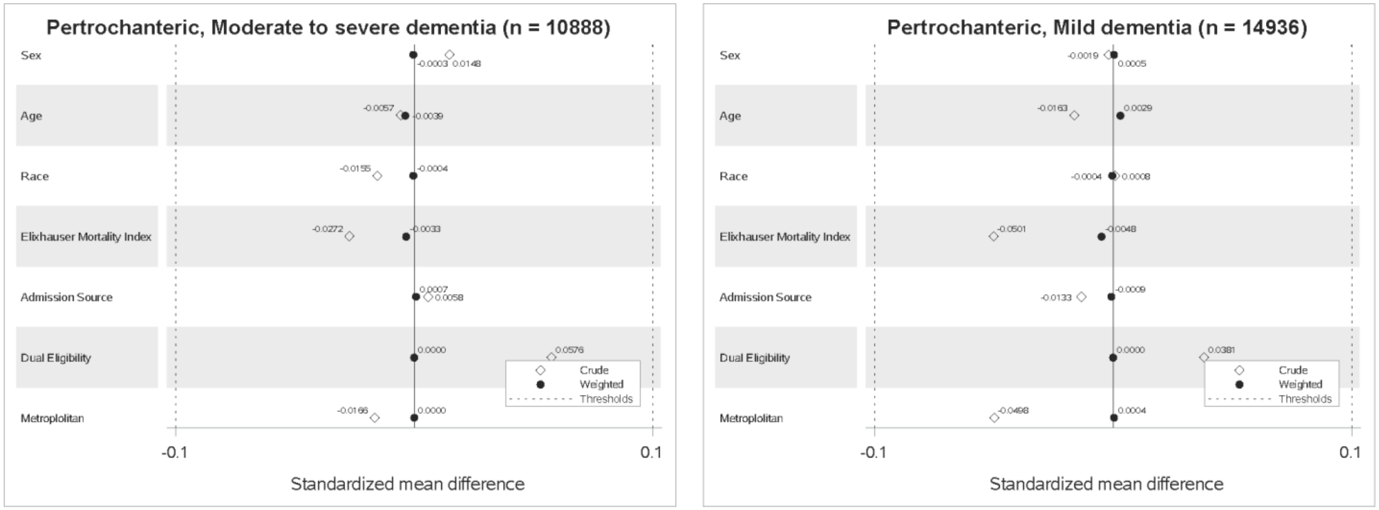

© 2024 Adler RR et al. *JAMA Network Open*.

eFigure 3. Covariate Balance Before and After IPTW for Patients With Subtrochanteric Fracture

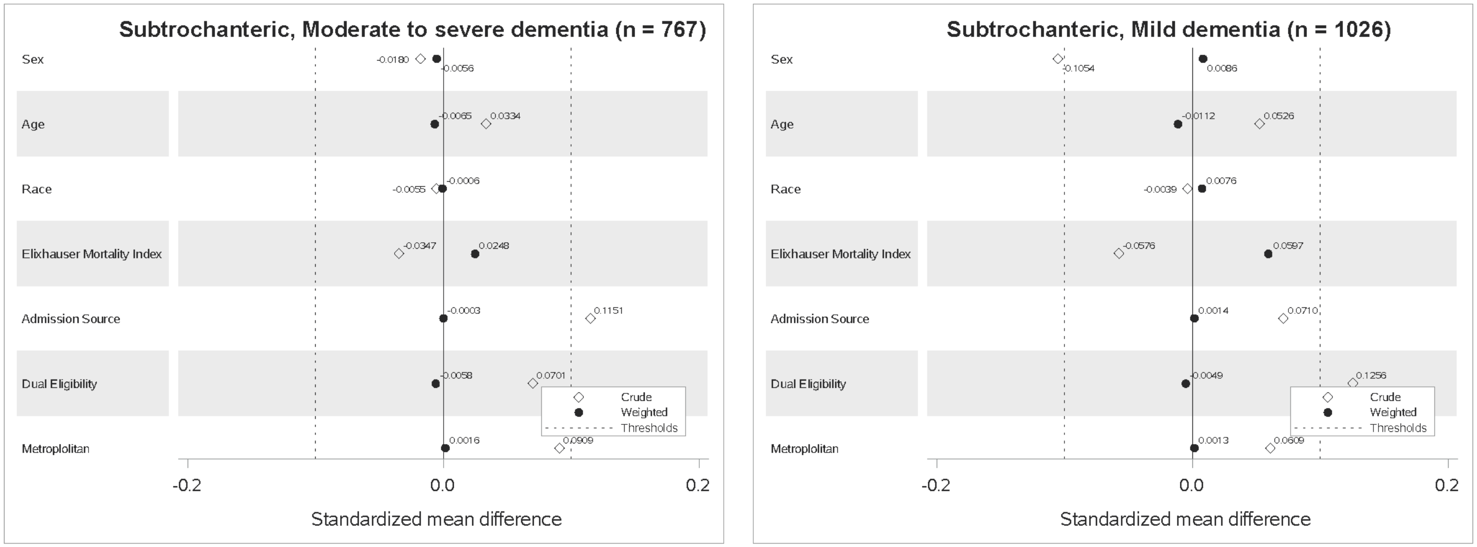

© 2024 Adler RR et al. *JAMA Network Open*.

eFigure 4. Covariate Balance Before and After IPTW for Patients With Fractures in Multiple Locations

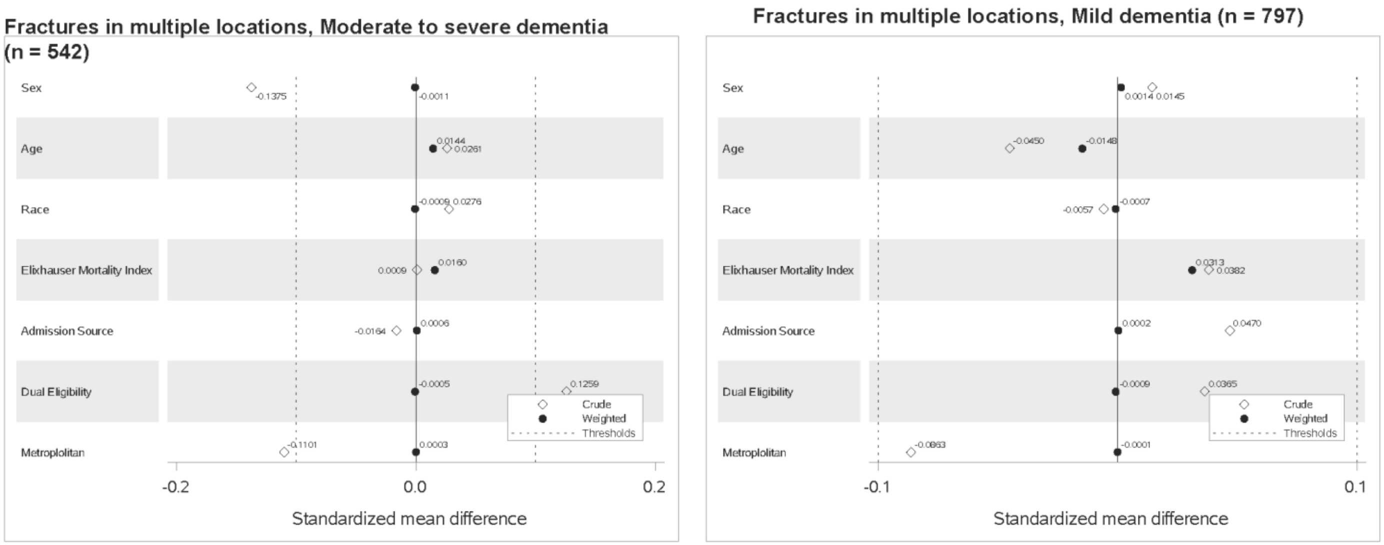

© 2024 Adler RR et al. *JAMA Network Open*.

eTable 1. Standardized Mean Difference for Crude and IPTW Data by Fracture Location

		Mild Dementia		Moderate to Severe Dementia	
**Fracture Location**	**Variable**	**Crude SMD**	**Weighted SMD**	**Crude SMD**	**Weighted SMD**
**Head and Neck of Femur**	Sex	-0.0513	-0.0039	-0.0221	-0.0016
	Age	-0.0147	-0.0075	-0.0393	-0.0048
	Race	0.0625	-0.0008	0.0893	-0.0065
	Elixhauser Mortality Index	-0.0682	0.0398	-0.1254	0.0064
	Admission Source	0.0027	-0.0060	0.0608	-0.0024
	Dual Eligibility	-0.0521	-0.0011	-0.0073	0.0045
	Metropolitan	-0.0034	0.0008	-0.0186	0.0069
**Pertrochanteric**	Sex	-0.0019	0.0005	0.0148	-0.0003
	Age	-0.0163	0.0029	-0.0057	-0.0039
	Race	0.0008	-0.0004	-0.0155	-0.0004
	Elixhauser Mortality Index	-0.0501	-0.0048	-0.0272	-0.0033
	Admission Source	-0.0133	-0.0009	0.0058	0.0007
	Dual Eligibility	0.0381	-0.0000	0.0576	-0.0000
	Metropolitan	-0.0498	0.0004	-0.0166	0.0000
**Subtrochanteric**	Sex	-0.1054	0.0086	-0.0180	-0.0056
	Age	0.0526	-0.0112	0.0334	-0.0065
	Race	-0.0039	0.0076	-0.0055	-0.0006
	Elixhauser Mortality Index	-0.0576	0.0597	-0.0347	0.0248
	Admission Source	0.0710	0.0014	0.1151	-0.0003
	Dual Eligibility	0.1256	-0.0049	0.0701	-0.0058
	Metropolitan	0.0609	0.0013	0.0909	0.0016
**Multiple Fracture Locations**	Sex	0.0145	0.0014	-0.1375	-0.0011
	Age	-0.0450	-0.0148	0.0261	0.0144
	Race	-0.0057	-0.0007	0.0276	-0.0009
	Elixhauser Mortality Index	0.0382	0.0313	0.0009	0.0160
	Admission Source	0.0470	0.0002	-0.0164	0.0006
	Dual Eligibility	0.0365	-0.0009	0.1259	-0.0005
	Metropolitan	-0.0863	-0.0001	-0.1101	0.0003

© 2024 Adler RR et al. *JAMA Network Open*.

eTable 2. Unadjusted and Adjusted Likelihood of Outcomes of Community-Dwelling People With Moderate to Severe Dementia Treated Surgically vs Nonsurgically, by Hip Fracture Location

**Outcome**	**Unadjusted**					**Adjusted[a]**				
	**Non-surgical Management n (%)**	**Surgical Management n (%)**	**OR**	**95% CI**	**p-value**	**Non-surgical Management n (%)**	**Surgical Management n (%)**	**OR**	**95% CI**	**p-value**
**Death**										
**Within 30 days**										
Fracture of head and neck of femur (S72.0)	380 (28.0)	1043 (10.8)	0.31	(0.27, 0.36)	<.001	369 (27.1)	1048 (10.9)	0.33	(0.29, 0.38)	<.001
Pertrochanteric fracture (S72.1)	1026 (13.4)	395 (12.3)	0.91	(0.80, 1.03)	.14	1020 (13.3)	400 (12.5)	0.93	(0.82, 1.05)	.25
Subtrochanteric fracture (S72.2)	72 (13.3)	34 (15.0)	1.15	(0.73, 1.80)	.56	71 (13.2)	35 (15.2)	1.18	(0.76, 1.84)	.47
Multiple locations	38 (13.2)	32 (12.6)	0.94	(0.57, 1.55)	.81	38 (13.2)	33 (12.9)	0.97	(0.59, 1.62)	.91
**Within 90 days**										
Fracture of head and neck of femur (S72.0)	513 (37.8)	2148 (22.3)	0.47	(0.42, 0.53)	<.001	499 (36.7)	2158 (22.4)	0.50	(0.44, 0.56)	<.001
Pertrochanteric fracture (S72.1)	1873 (24.4)	741 (23.1)	0.93	(0.85, 1.03)	.15	1864 (24.3)	750 (23.4)	0.95	(0.86, 1.05)	.31
Subtrochanteric fracture (S72.2)	135 (25.0)	58 (25.6)	1.03	(0.72, 1.47)	.87	134 (24.8)	59 (26.2)	1.08	(0.75, 1.54)	.69
Multiple locations	68 (23.7)	53 (20.8)	0.85	(0.56, 1.27)	.41	69 (23.9)	54 (21.1)	0.85	(0.56, 1.28)	.43
**Within 180 days**										
Fracture of head and neck of femur (S72.0)	620 (45.7)	3063 (31.8)	0.56	(0.49, 0.62)	<.001	602 (44.3)	3075 (31.9)	0.59	(0.53, 0.66)	<.001
Pertrochanteric fracture (S72.1)	2572 (33.5)	1047 (32.6)	0.96	(0.88, 1.05)	.39	2562 (33.4)	1057 (33.0)	0.98	(0.90, 1.07)	.69
Subtrochanteric fracture (S72.2)	181 (33.5)	85 (37.4)	1.19	(0.86, 1.64)	.29	180 (33.4)	86 (37.9)	1.21	(0.88, 1.68)	.24
Multiple locations	89 (31.0)	78 (30.6)	0.98	(0.68, 1.41)	.91	89 (31.2)	79 (30.9)	0.99	(0.68, 1.43)	.95
**In-Hospital Delirium**										
Fracture of head and neck of femur (S72.0)	227 (16.7)	1787 (18.6)	1.14	(0.97, 1.33)	.11	214 (15.7)	1800 (18.7)	1.23	(1.06, 1.44)	.008
Pertrochanteric fracture (S72.1)	1304 (17.0)	548 (17.1)	1.01	(0.90, 1.13)	.90	1300 (16.9)	552 (17.2)	1.02	(0.92, 1.14)	.71
Subtrochanteric fracture (S72.2)	92 (17.0)	48 (21.2)	1.31	(0.88, 1.93)	.18	91 (16.9)	48 (21.3)	1.32	(0.90, 1.96)	.16
Multiple locations	60 (20.9)	38 (14.9)	0.66	(0.43, 1.03)	.07	60 (20.9)	38 (14.9)	0.66	(0.42, 1.04)	.07
**Hospice Referral[b]**										
Fracture of head and neck of femur (S72.0)	476 (38.1)	2749 (29.2)	0.67	(0.59, 0.76)	<.001	471 (37.4)	2755 (29.3)	0.69	(0.61, 0.79)	<.001
Pertrochanteric fracture (S72.1)	2171 (29.4)	883 (28.4)	0.95	(0.87, 1.04)	.29	2162 (29.3)	889 (28.6)	0.97	(0.88, 1.06)	.48
Subtrochanteric fracture (S72.2)	150 (28.9)	57 (26.2)	0.87	(0.62, 1.23)	.44	150 (28.8)	58 (26.7)	0.90	(0.63, 1.29)	.58
Multiple locations	67 (24.6)	74 (29.8)	1.30	(0.89, 1.90)	.17	68 (24.9)	75 (30.1)	1.30	(0.88, 1.91)	.19

© 2024 Adler RR et al. *JAMA Network Open*.

**Home Health Care**[c]										
Fracture of head and neck of femur (S72.0)	70 (6.6)	480 (5.4)	0.80	(0.61, 1.04)	.10	71 (6.6)	480 (5.4)	0.80	(0.62, 1.04)	.10
Pertrochanteric fracture (S72.1)	301 (4.3)	123 (4.2)	0.96	(0.77, 1.19)	.70	301 (4.3)	123 (4.2)	0.96	(0.77, 1.19)	.70
Subtrochanteric fracture (S72.2)	27 (5.5)	NA[d]	0.33	(0.12, 0.94)	.04	27 (5.5)	NA[d]	0.32	(0.11, 0.94)	.04
Multiple locations	14 (5.3)	NA[d]	0.72	(0.30, 1.71)	.46	14 (5.5)	NA[d]	0.74	(0.31, 1.76)	.50

Medicare Patients 2017-2018 Among Patients With Moderate to Severe Dementia

[a]Inverse propensity weighted regression. For each hip fracture location and CFI severity group combination, propensity scores for the management groups were calculated by age, sex, race, elixhauser mortality index, admission source, urbanicity, and dual eligibility, respectively.

[b]Restricted to patients discharged alive.

[c]Restricted to patients who were discharged alive and didn't die within 10 days after discharge.

[d]Data suppressed according to Medicare data policies.

© 2024 Adler RR et al. *JAMA Network Open*.

eTable 3. Unadjusted and Adjusted Likelihood of Outcomes of Community-Dwelling People Mild With Dementia Treated Surgically vs Nonsurgically, by Hip Fracture Location

Outcome	Unadjusted					Adjusted[a]				
	Non-surgical Management n (%)	Surgical Management n (%)	OR	95% CI	p-value	Non-surgical Management n (%)	Surgical Management n (%)	OR	95% CI	p-value
**Death**										
**Within 30 days**										
Fracture of head and neck of femur (S72.0)	319 (19.8)	1216 (8.3)	0.37	(0.32, 0.42)	<.001	306 (19.0)	1221 (8.3)	0.39	(0.34, 0.45)	<.001
Pertrochanteric fracture (S72.1)	1041 (10.0)	412 (9.1)	0.91	(0.80, 1.02)	.11	1032 (9.9)	419 (9.3)	0.93	(0.83, 1.05)	.25
Subtrochanteric fracture (S72.2)	79 (11.4)	33 (9.9)	0.85	(0.56, 1.30)	.46	78 (11.3)	34 (10.1)	0.88	(0.57, 1.35)	.55
Multiple locations	48 (10.2)	39 (12.0)	1.20	(0.77, 1.89)	.42	48 (10.2)	39 (12.2)	1.22	(0.78, 1.91)	.39
**Within 90 days**										
Fracture of head and neck of femur (S72.0)	459 (28.4)	2685 (18.3)	0.57	(0.50, 0.64)	<.001	443 (27.4)	2692 (18.4)	0.60	(0.53, 0.67)	<.001
Pertrochanteric fracture (S72.1)	2088 (20.0)	845 (18.7)	0.92	(0.84, 1.00)	.06	2073 (19.9)	859 (19.0)	0.95	(0.87, 1.03)	.22
Subtrochanteric fracture (S72.2)	154 (22.3)	61 (18.3)	0.78	(0.56, 1.09)	.15	153 (22.1)	62 (18.6)	0.80	(0.58, 1.12)	.20
Multiple locations	102 (21.6)	67 (20.6)	0.94	(0.67, 1.32)	.73	102 (21.6)	67 (20.8)	0.95	(0.67, 1.35)	.78
**Within 180 days**										
Fracture of head and neck of femur (S72.0)	563 (34.9)	3879 (26.5)	0.67	(0.60, 0.76)	<.001	547 (33.9)	3888 (26.5)	0.71	(0.63, 0.79)	<.001
Pertrochanteric fracture (S72.1)	2939 (28.2)	1233 (27.3)	0.96	(0.89, 1.03)	.26	2922 (28.0)	1251 (27.7)	0.98	(0.91, 1.06)	.69
Subtrochanteric fracture (S72.2)	214 (30.9)	75 (22.5)	0.65	(0.48, 0.88)	.006	212 (30.7)	75 (22.5)	0.66	(0.48, 0.89)	.007
Multiple locations	148 (31.4)	102 (31.4)	1.00	(0.74, 1.35)	>.99	148 (31.4)	102 (31.5)	1.00	(0.74, 1.36)	.99
**In-Hospital Delirium**										
Fracture of head and neck of femur (S72.0)	243 (15.1)	2242 (15.3)	1.02	(0.89, 1.17)	.78	225 (13.9)	2257 (15.4)	1.12	(0.97, 1.30)	.11
Pertrochanteric fracture (S72.1)	1577 (15.1)	677 (15.0)	0.99	(0.90, 1.09)	.84	1563 (15.0)	693 (15.4)	1.03	(0.93, 1.13)	.58
Subtrochanteric fracture (S72.2)	108 (15.6)	51 (15.3)	0.97	(0.67, 1.41)	.89	105 (15.2)	56 (16.8)	1.13	(0.78, 1.62)	.53
Multiple locations	83 (17.6)	52 (16.0)	0.89	(0.60, 1.32)	.57	83 (17.6)	53 (16.2)	0.91	(0.62, 1.33)	.62
**Hospice Referral[b]**										
Fracture of head and neck of femur (S72.0)	410 (26.8)	3398 (23.6)	0.84	(0.74, 0.95)	.007	405 (26.3)	3400 (23.6)	0.86	(0.77, 0.97)	.02
Pertrochanteric fracture (S72.1)	2462 (24.1)	968 (21.9)	0.88	(0.81, 0.96)	.004	2449 (24.0)	980 (22.2)	0.91	(0.83, 0.99)	.02
Subtrochanteric fracture (S72.2)	158 (23.7)	58 (17.7)	0.70	(0.50, 0.97)	.03	157 (23.5)	58 (17.7)	0.70	(0.50, 0.98)	.04
Multiple locations	115 (25.3)	78 (24.5)	0.96	(0.70, 1.32)	.79	115 (25.2)	79 (24.7)	0.97	(0.70, 1.36)	.87
**Home Health Care[c]**										

© 2024 Adler RR et al. *JAMA Network Open*.

Fracture of head and neck of femur (S72.0)	122 (8.8)	974 (7.0)	0.78	(0.64, 0.95)	.01	123 (8.8)	972 (7.0)	0.78	(0.64, 0.95)	.01
Pertrochanteric fracture (S72.1)	564 (5.8)	227 (5.3)	0.92	(0.79, 1.08)	.31	565 (5.8)	226 (5.3)	0.92	(0.78, 1.07)	.28
Subtrochanteric fracture (S72.2)	23 (3.6)	12 (3.9)	1.08	(0.53, 2.20)	.83	23 (3.6)	13 (4.2)	1.18	(0.57, 2.42)	.66
Multiple locations	23 (5.3)	15 (4.8)	0.92	(0.48, 1.76)	.81	23 (5.3)	15 (4.8)	0.90	(0.46, 1.77)	.77

Medicare Patients 2017-2018 Among Patients With Mild Dementia1

[a]Inverse propensity weighted regression. For each hip fracture location and CFI severity group combination, propensity scores for the management groups were calculated by age, sex, race, elixhauser mortality index, admission source, urbanicity, and dual eligibility, respectively.

[b]Restricted to patients discharged alive

[c]Restricted to patients who were discharged alive and didn't die within 10 days after discharge.

© 2024 Adler RR et al. *JAMA Network Open*.

eTable 4. Unadjusted and Adjusted Likelihood of Outcomes of Community-Dwelling People with Dementia Treated Surgically vs Nonsurgically, by Hip Fracture Location and Dementia Severity

	Moderate to Severe Dementia[1]						Mild Dementia[1]					
Outcome	Unadjusted			Adjusted[a]			Unadjusted			Adjusted[a]		
	Non-surgical Management	Surgical Management	p-value	Non-surgical Management	Surgical Management	p-value	Non-surgical Management	Surgical Management	p-value	Non-surgical Management	Surgical Management	p-value
**30-day Intensive Intervention[b]**												
Fracture of head and neck of femur (S72.0)	4%	4%	.94	4%	4%	.39	3%	2%	.93	2%	2%	.49
Pertrochanteric fracture (S72.1)	4%	4%	.88	4%	4%	.84	2%	2%	.82	2%	2%	.64
Subtrochanteric fracture (S72.2)	4%	4%	.63	4%	4%	.63	3%	2%	.19	3%	2%	.18
Multiple locations	3%	4%	.07	3%	4%	.05	1%	3%	.24	1%	2%	.26
**90-day Intensive Intervention[b]**												
Fracture of head and neck of femur (S72.0)	6%	6%	.94	5%	6%	.39	4%	3%	.93	3%	3%	.49
Pertrochanteric fracture (S72.1)	5%	5%	.88	5%	5%	.84	3%	3%	.82	3%	3%	.64
Subtrochanteric fracture (S72.2)	4%	4%	.63	4%	4%	.63	4%	3%	.19	4%	3%	.18
Multiple locations	3%	5%	.07	3%	5%	.05	2%	4%	.24	2%	4%	.26
**180-day Intensive Intervention[b]**												
Fracture of head and neck of femur (S72.0)	NA[c]	7%	.94	NA[c]	7%	.39	NA[c]	NA[c]	.93	NA[c]	NA[c]	.49
Pertrochanteric fracture (S72.1)	NA[c]	NA[c]	.88	NA[c]	NA[c]	.84	4%	NA[c]	.82	4%	NA[c]	.64
Subtrochanteric fracture (S72.2)	NA[c]	NA[c]	.63	NA[c]	NA[c]	.63	NA[c]	NA[c]	.19	NA[c]	NA[c]	.18
Multiple locations	NA[c]	NA[c]	.07	NA[c]	NA[c]	.05	NA[c]	NA[c]	.24	NA[c]	NA[c]	.26
**30-day Admission to Skilled Nursing Facility[b]**												
Fracture of head and neck of femur (S72.0)	49%	65%	<.001	50%	65%	<.001	56%	64%	<.001	56%	64%	<.001
Pertrochanteric fracture (S72.1)	64%	67%	<.001	64%	67%	<.001	65%	67%	<.001	65%	67%	<.001
Subtrochanteric fracture (S72.2)	66%	69%	.10	66%	68%	.12	64%	73%	.07	64%	73%	.12
Multiple locations	65%	70%	.23	65%	70%	.29	67%	68%	.92	67%	68%	.85
**90-day Admission to Skilled Nursing Facility[b]**												

© 2024 Adler RR et al. *JAMA Network Open*.

Fracture of head and neck of femur (S72.0)	51%	66%	<.001	51%	66%	<.001	58%	65%	<.001	58%	65%	<.001
Pertrochanteric fracture (S72.1)	65%	68%	<.001	65%	68%	<.001	67%	69%	<.001	67%	68%	<.001
Subtrochanteric fracture (S72.2)	NA[c]	NA[c]	.10	NA[c]	NA[c]	.12	NA[c]	75%	.07	NA[c]	74%	.12
Multiple locations	66%	72%	.23	66%	72%	.29	68%	70%	.92	68%	70%	.85
**180-day Admission to Skilled Nursing Facility[b]**											
Fracture of head and neck of femur (S72.0)	NA[c]	67%	<.001	NA[c]	67%	<.001	NA[c]	NA[c]	<.001	NA[c]	NA[c]	<.001
Pertrochanteric fracture (S72.1)	NA[c]	69%	<.001	NA[c]	69%	<.001	NA[c]	NA[c]	<.001	NA[c]	NA[c]	<.001
Subtrochanteric fracture (S72.2)	NA[c]	NA[c]	.10	NA[c]	NA[c]	.12	NA[c]	NA[c]	.07	NA[c]	NA[c]	.12
Multiple locations	NA[c]	NA[c]	.23	NA[c]	NA[c]	.29	NA[c]	NA[c]	.92	NA[c]	NA[c]	.85
**30-day Admission to Nursing Home[b]**											
Fracture of head and neck of femur (S72.0)	20%	22%	.17	21%	22%	.23	21%	20%	.98	21%	20%	.67
Pertrochanteric fracture (S72.1)	22%	23%	.33	22%	23%	.75	21%	22%	.10	21%	22%	.29
Subtrochanteric fracture (S72.2)	24%	30%	.06	24%	30%	.11	24%	20%	.14	24%	19%	.06
Multiple locations	23%	24%	.37	23%	24%	.65	22%	25%	.10	22%	25%	.14
**90-day Admission to Nursing Home[b]**											
Fracture of head and neck of femur (S72.0)	24%	25%	.17	24%	25%	.23	23%	23%	.98	23%	23%	.67
Pertrochanteric fracture (S72.1)	25%	26%	.33	25%	26%	.75	24%	25%	.10	24%	25%	.29
Subtrochanteric fracture (S72.2)	27%	34%	. 06	27%	33%	.11	26%	25%	.14	26%	24%	.06
Multiple locations	26%	29%	.37	27%	28%	.65	24%	29%	.10	24%	28%	.14
**180-day Admission to Nursing Home[b]**											
Fracture of head and neck of femur (S72.0)	NA[c]	27%	.17	NA[c]	27%	.23	NA[c]	25%	.98	NA[c]	25%	.67
Pertrochanteric fracture (S72.1)	NA[c]	28%	.33	NA[c]	28%	.75	26%	NA[c]	.10	26%	NA[c]	.29
Subtrochanteric fracture (S72.2)	NA[c]	NA[c]	. 06	NA[c]	NA[c]	.11	NA[c]	NA[c]	.14	NA[c]	NA[c]	.06
Multiple locations	NA[c]	NA[c]	.37	NA[c]	NA[c]	.65	NA[c]	NA[c]	.10	NA[c]	NA[c]	.14
**30-day Readmission to Acute Care Facility[b]**											
Fracture of head and neck of femur (S72.0)	14%	17%	<.001	14%	17%	<.001	15%	13%	.40	15%	13%	.77
Pertrochanteric fracture (S72.1)	14%	15%	.89	14%	15%	.94	13%	13%	.63	13%	13%	.75
Subtrochanteric fracture (S72.2)	16%	17%	.97	16%	17%	.98	13%	14%	.38	13%	14%	.46
Multiple locations	18%	12%	.12	18%	13%	.11	13%	18%	0.05	13%	18%	.06

© 2024 Adler RR et al. *JAMA Network Open*.

**90-day Readmission to Acute Care Facility[b]**												
Fracture of head and neck of femur (S72.0)	25%	31%	<.001	25%	31%	<.001	25%	24%	.40	24%	24%	.77
Pertrochanteric fracture (S72.1)	28%	27%	.89	28%	27%	.94	23%	23%	.63	23%	23%	.75
Subtrochanteric fracture (S72.2)	29%	30%	.97	29%	30%	.98	24%	23%	.38	24%	23%	.46
Multiple locations	28%	24%	.12	29%	25%	.11	22%	28%	0.05	22%	28%	.06
**180-day Readmission to Acute Care Facility[b]**												
Fracture of head and neck of femur (S72.0)	33%	40%	<.001	33%	40%	<.001	33%	32%	.40	32%	32%	.77
Pertrochanteric fracture (S72.1)	37%	37%	.89	37%	37%	.94	31%	30%	.63	31%	30%	.75
Subtrochanteric fracture (S72.2)	NA[c]	39%	.97	NA[c]	39%	.98	NA[c]	NA[c]	.38	NA[c]	NA[c]	.46
Multiple locations	NA[c]	NA[c]	.12	NA[c]	NA[c]	.11	29%	NA[c]	0.05	29%	NA[c]	.06

Medicare Patients 2017-2018

[a]Inverse propensity weighted regression. For each hip fracture location and CFI severity group combination, propensity scores for the management groups were calculated by age, sex, race, elixhauser mortality index, admission source, urbanicity, and dual eligibility, respectively.

[b]Restricted to patients discharged alive

[c] NA = Not Applicable; No outcome event was observed on or after the last day of the follow-up period.

© 2024 Adler RR et al. *JAMA Network Open*.

eReference

1. Park CM, Sison SDM, McCarthy EP, et al. Claims-Based Frailty Index as a Measure of Dementia Severity in Medicare Claims Data. *The Journals of Gerontology: Series A*. Published online July 10, 2023:glad166. doi:10.1093/gerona/glad166

© 2024 Adler RR et al. *JAMA Network Open*.
