# Supplementary material for: Hip Fracture Treatment and Outcomes Among Community-Dwelling People Living With Dementia
Source: JAMA Netw Open. 2024 May 30;7(5):e2413878. doi: 10.1001/jamanetworkopen.2024.13878 (PMC11140536; doi:10.1001/jamanetworkopen.2024.13878)
Supplement: Supplement 2. — Data Sharing Statement [file jamanetwopen-e2413878-s002.pdf]

Data Sharing Statement

Adler. Hip Fracture Treatment and Outcomes Among Community-Dwelling People Living With Dementia. *JAMA Netw Open*. Published May 30, 2024.
doi:10.1001/jamanetworkopen.2024.13878

Data
Data available: No

Additional Information
Explanation for why data not available: We are unable to share individual patient data because the data use agreement with CMS restricts us from doing so.
